# Consumers’ Health and Environmental Attitudes and Local Food Purchases

**DOI:** 10.3390/ijerph22020298

**Published:** 2025-02-17

**Authors:** Lan Tran, Ye Su

**Affiliations:** 1 Cooperative Research and Extension, College of Agriculture, Environment and Human Science, Lincoln University of Missouri, 311 Foster Hall, Jefferson City, MO 65101, USA; tranl@lincolnu.edu; 2 International Business and Economics, VNU-University of Economics and Business, 144 Xuan Thuy, Hanoi 1000, Vietnam

**Keywords:** local food, consumer preferences, SEM, health, environment

## Abstract

There has been increasing interest in the health and environmental benefits of the growth of local food, especially since the COVID-19 pandemic. In the United States, local food and labels have many implications and attributes, such as organic, non-GMO, and reduced-chemical production. Therefore, consumers’ purchase decisions and willingness to pay for local labels with sustainable attributes are heterogeneous. This study uses a consumer survey in Missouri to examine how differences and differentiations in health and environmental attitudes affect consumers’ willingness to pay for local food. A discrete choice experiment and a structural equation model (SEM) were employed to measure how personal attitudes affect consumer’s willingness to pay for local labels (typical label and state-grown). Results show that supportive attitudes toward local farms and farmers positively affect consumer preferences for locally labeled produce, but the premiums will be lower if they are more concerned about GMOs and pesticide residue in food. No evidence was found for the effects of general environmental attitudes on willingness to pay for local food.

## 1. Introduction

Consumption of local food has increased remarkably over the past decade. Sales of local food overall increased from USD 6 billion to USD 9 billion between 2012 and 2020 in the United States [1]. There has been growing interest in investigating the factors that contribute to the growth of local food purchase [2,3,4,5,6]. Several studies have reported that consumers’ beliefs and attitudes can affect purchase decisions and price premiums for locally grown food [7,8,9]. Nevertheless, the effects of environmental and health attitudes on consumer preferences for local food products have been overlooked. It is unclear how changes or differences in health consciousness and environmental attitudes influence consumer willingness to pay (WTP) for local attributes.

The concept of local food is not explicitly related to health or environmental benefits. In the literature, “local food” is mostly related to the geographic proximity of production and consumption, e.g., the distance between the food produced and consumed or “food miles” [10,11]. Despite differences in the acceptable distance of localness, other characteristics such as freshness, healthfulness, safety, high quality, traceability, and economic and environmental benefits to local communities are attributed by consumers [12,13,14]. Further, local food may be referred to as organic [15,16] or sustainable food [17,18]. Regardless of the variations and the ambiguity in the definition of local food, numerous empirical studies have observed differences in consumers’ WTP and sources of heterogeneity in their preferences for local food [19]. However, little research has examined the role of different attitudes in valuing local food’s environmental and health characteristics.

With growing interest in the health dimension and environmental awareness, especially since the COVID-19 pandemic, it is worth considering the determinants of motivation for local food purchases, particularly concerning health and environmental attitudes. As reported by recent studies [19,20], a large majority of studies (about 85% [19]) use discrete choice modeling to examine consumer WTP and factors affecting the WTP for local labels. While several measurable factors like age, education, and income are significant determinants of WTP, little is known about the effects of unobservable consumer attitudes because of the difficulties in incorporating these variables into the choice experiments. On the other hand, if influences of consumer attitudes are revealed through health and environmental features, there would be required reductions in the number of attributes and attribute levels given the complexity of a choice experiment. Apparently, this might cause variations and potential bias in the WTP estimation.

To solve these potential issues, we use two-stage analyses. First, we estimate individual consumer WTP for “local” food using a mixed logit approach based on a choice experiment. Second, we apply linear structural equation models to measure the influences of the latent variables of health consciousness and environmental attitudes on consumer WTP. We hypothesize that an increase in health concerns regarding GMOs and pesticide residue leads to higher consumer WTP for local food. Similarly, we expect a positive relationship between environmental concerns and WTP for local food, where individuals with stronger environmental concern are willing to pay a higher premium. However, we anticipate that the impact of health and environmental concerns on consumer WTP will be relatively smaller compared to the influence of factors such as support for local farms and community attitudes. Furthermore, these relationships may be moderated by the availability of organic food options.

The study contributes to the literature on local food in the following aspects. First, it adds knowledge about how health consciousness and environmental awareness influence local food consumption. Second, it provides a better understanding of the effects of consumer attitudes on the premium for “local” labels. This can help local food producers choose appropriate production methods to improve their business by targeting profitable niche markets and serving consumers better. Third, it explores the ability to evaluate the effects of latent variables on the WTP measurement based on a choice experiment. Finally, the results of the study can be used by policymakers and other stakeholders like the Missouri Grown program to improve local food sales, support low-income local food eaters, and address health and environmental concerns in Missouri.

## 2. Literature Review

### 2.1. Local Food Consumption and Its Definitions

Local food consumption has been growing in the U.S. since the 1990s. Starting with the Society for Nutrition Education’s 1981 guidelines, healthy and local food movements have been widespread all over the U.S. [21]. Under their guidelines, consumers are encouraged to build healthy eating habits and consume locally grown food. Some evidence has shown that even though consumers do not actively shop for them initially, they show greater preferences for “local” foods during shopping [2,22,23,24,25,26,27]. According to the U.S. Department of Agriculture, National Agricultural Statistics Service (USDA, NASS), direct-to-consumer sales amounted to USD 11.8 billion in 2017, which increased to USD 17.5 billion in 2022 (an annual real growth rate of 4.6 percent), compared with USD 1.2 billion in 2007 and USD 551 million in 1997 [1,10,28,29].

The expansion of community-supported agriculture (CSA), farmers’ markets (FMs), and other forms of direct marketing practices are meaningful indicators of the growth of local food. The number of FMs increased from 1755 in 1994 to 8720 in 2020, and CSAs increased from one in 1984 to an estimated 5638 in 2015 [30]. Also, the development of farm-to-school (FTS), farm-to-restaurant, and farm-to-institute (hospitals or government organizations) programs that use local farms as food suppliers significantly contribute to the growth of local food consumption.

While local food has become so important, there is no universal definition for local food [10,11]. In the literature, the definitions for local food vary in terms of how far the food is grown or sold from the consumer location, how naturally the food is produced (like pesticide-free, hormone-free, or organic food, etc.), and whether the food is produced and sold by small and family farms [31]. However, there is no consensus for a specific mileage distance between where the food is produced and consumed for the “local” concept. Some consumers use shorter distances, like 25 or 50 miles, while others consider their own or surrounding counties, compared to 400 miles or state boundaries as suggested by the 2008 Farm Bill. Consumers living in small cities or towns are likely to use 50 miles to define local food while those in large metropolitan areas consider a 100-mile distance [32]. The lack of clarity in the “local” concept potentially makes the benefits of local food become unguaranteed and different to the buyers and other stakeholders.

### 2.2. Consumer Motivations for Local Food and the Role of Health and Environmental Attitudes

Despite various definitions, consumers have shown consistent motivations toward local food purchases [12,33]. Past surveys have indicated that the top reasons for buying “local” food are the freshness and taste of the products and an awareness or belief of economic benefits to the local community [34,35]. For example, according to the 2014 FMI’s U.S. Grocery Shopper Trends Survey, 86% of consumers cited freshness as the most important reason for local food purchase, followed by supporting the local economy (75%), and better taste (61%), respectively [34].

Freshness and taste can be expected by the nature of the “shorter distance” between consumers and producers regarding locally produced food. Compared to the produce that has been shipped from far away, locally grown options are generally fresher. Regarding the local community, the economic benefits of local food can be explained in multiple ways. First, the money spent on local food is more likely to be circulated within the local community because farmers and other food businesses buy locally, hire local employees, and work with other local businesses [36]. By removing “middleman” agents, the local food system benefits farmers by allowing them to obtain a greater share of the food dollar. Additionally, direct marketing practices, which have often been considered the main local food marketing channel, provide market access for new and beginning farmers, especially with government support for the development of farmers’ markets [35].

How do health and environmental attitudes affect consumers’ motivations for local food? Local food is often assumed to be environmentally beneficial because of reduced transportation emissions and output (e.g., carbon dioxide) [37]. In addition, local producers may use input from suppliers nearby for their productions, resulting in smaller carbon footprints. Further, local food is usually found to be associated with organic or environmentally friendly production methods; e.g., reduced or chemical-free produce is often sold at FMs [30]. However, according to a longitudinal study by Schoolman [38], the magnitude of the association between direct market and decreased on-farm use of agricultural chemicals is weak. On the other hand, while producing and eating local food may have positive effects on the environment, there are no direct comparisons to non-local foods. Overall, there is little evidence of whether local food systems are more environmentally friendly than regional, national, or global food systems [14].

The influence of health attitudes on local food consumption is inadequately addressed in the literature. Discussions about the relationship between personal health and local food are related to freshness, health risks, and healthy behaviors. First, locavores find locally produced food fresher and then possibly healthier than non-local ones. This could be true, especially for vegetables and fruits harvested when they are ripe and travel shorter distances [39]. However, whether fresher products can be considered healthier is arguable, and this can differ significantly across types of food. In addition, while nutrition is important for health and development, freshness is not necessarily an indicator of the nutritional value of a food product. For example, frozen and canned corn are as nutritious as fresh corn, while canned tomatoes provide a little bit lower nutrient intake (nutrient content per calorie) than fresh tomatoes, but at a substantially lower cost [40].

Second, as previously addressed, vegetables and fruits provided by local farmers are generally non-GMO-produced with reduced, free chemicals, or even organically produced. Buyers may also be informed about other stories regarding production as they have more chances to communicate directly with the providers via direct marketing practices. In this regard, local food choice is probably preferred for lower health risks regarding pesticide residue and GMO issues. Third, eating local food may develop and improve healthy behaviors. Eating well is more and more important for good health and nutrition. Consuming more fruits and vegetables in a daily diet can reduce the risk of heart disease and some forms of cancer, and lower rates of diabetes and body mass index [41]. Research has shown that individuals who frequently consume vegetables and fruits and eat a greater variety in their diet may have health interests and healthy eating habits [5,42]. Local produce is a good option for healthy food choices, and vegetables and fruits are indeed the most popular products to buy locally [30]. However, as in the case of environmental attitudes, there are no direct comparisons of the effects of health attitudes on the purchase of local and non-local options, though some studies use eating imported organic food as a proxy for healthy eating behavior.

### 2.3. Potential Factors Affecting WTP for Local Food

Numerous studies have shown that consumers who prefer local food over non-local food are willing to pay a premium for local attributes [27,43,44,45,46,47,48]. However, the literature on consumer preferences has also indicated variations in the WTP for local food [19]. Given different definitions of “local” and various local labels, it would not be surprising. The reasons for the variations include differences in study designs, selected products, locations or regions, and analytic methods. In addition, heterogeneity in preferences for local food has been addressed in the literature. Among sources of heterogeneity, the socio-demographic characteristics of the relevant survey participants may affect the WTP estimate.

Several studies have presented that female, older, higher educated, or higher income consumers were willing to pay extra for local food. They also showed that those who were connected to agriculture (e.g., raised on a farm, living in rural areas) or those who supported local communities, small and family farms, or environmentally friendly practices were often willing to pay higher premiums for local products [12,47,49,50]. It would be worth noting that the effects of consumer characteristics are not consistent across previous studies. Apart from gender, the effects of age, education, and income are not significant among meta-analysis reviews on the WTP for local food [19,51].

The influences of health and environmental attitudes on the WTP for local labels are debatable. For example, pro-environmental attitudes regarding reduced greenhouse gasses can lead to significantly higher WTP for food traveling shorter distances [17]. However, the higher WTP depends on product type [52]. Though several studies have shown a positive correlation between local food purchases and consumer health interests, no causal research has been found [19]. The effect of health attitude claims on the WTP for local food is understudied in the literature.

## 3. Methodology

### 3.1. Data

An online consumer survey for local food was conducted from November 2021 to March 2022 in Missouri, USA. The participants were required to be (1) the primary grocery shoppers of their households, (2) residents of Missouri, and (3) at least 18 years old and voluntarily participated in the survey. The survey was approved by the IRB board of Lincoln University of Missouri (approval # IRB F2020-01). In the survey questionnaire, respondents were asked to present their demographics, perceptions, attitudes, and shopping behaviors on fresh produce in the context of local and Missouri Grown brand food. Additionally, the respondents revealed their preferences for the local attributes of tomato purchase options through a choice experiment embedded within the survey [53]. In the experiment, “local” tomatoes were defined by carrying a “local” label or a state brand (Missouri Grown in this case). Tomato purchase options differed in four attributes: production method (organic, reduced pesticide use, or conventional), producer characteristics (small and medium family farms, large family farms, or large corporation), product label (local, Missouri Grown, not local or Missouri Grown), and price ($1.99, $2.99, or $3.99/lb.). Nine scenarios were randomly displayed to the respondents. The consumers stated their preferences for one pound (lb.) of different tomato profiles or chose to opt out of purchase (see Table 1 for an example).

Which choice for buying tomatoes would you prefer?
○ Option A○ Option B○ Option C○ None of them

A sample of 511 respondents were recruited through Amazon’s Mechanical Turk (MTurk), an online survey platform, for the survey. We used the Qualtrics platform to design questions, deliver the questionnaires, and manage the survey data. After removing the respondents who were not qualified for the requirements, had missing values, or did not meet the traditional criteria for an online survey (duplicated responses, bot detection, attention check, etc.), a sample of 352 usable responses was obtained for the study.

Table 2 reports the summary statistics of the sample. A slight majority of respondents were female (54.0%), which is expected for grocery shoppers. Participants’ ages ranged from 18 to 85, with an average age of 39.6 years old. Their average annual income was approximately USD 62,000. In general, the sample’s age and income statistics are comparable with that of the Missouri population, which has an average age of 38.8 and an average household income of USD 61,043 [54]. About three-quarters of the respondents were white (77.3%), which is slightly lower than the number of Caucasians in Missouri (80.3%) [54]. The respondents were largely well educated, with 42.6% having bachelor’s degrees and 20.7% with graduate/professional degrees. About two-thirds were living in suburban and rural Missouri. Half of the sample had at least one child under 17 years old in their family. Most respondents purchased local food in the past 12 months (85.5%). A majority of the sample bought fresh produce at least once a week (80.1%).

Table 3 reports descriptive results for the attitude variables of interest in the study. There are eleven consumer attitudes, including those related to sustainable community development, environmental protection, and concerns regarding GMOs and pesticide residue on food. The eleven indicators were measured using a 5-point Likert scale. The attitude instruments mostly have means from 3.2 to 4.2, with an SD of approximately 1.0–1.5, indicating different attitude levels (Table 3).

### 3.2. Empirical Models

This study investigates the influence of consumers’ attitudes towards health and the environment on their preferences for local food. It includes two stages. First, we characterize consumer preferences for local attributes using a choice modeling approach. Second, we estimate and evaluate the effects of health and environmental attitudes on the derived measurement of consumer preferences via linear regression models. Individual consumers’ WTPs for local attributes and proxies for the preferences are obtained by a discrete choice experiment (DCE) of tomato purchases (Supplementary Table S1).

In the first stage, the choice model of participant “i” chooses option or alternative “j” in the choice task “t” given alternative characteristics “x” is:

(1) $$P\left(y_{i}=j|x_{i},\beta \right)=\prod_{t=1}^{T}\left[\frac{exp\left({\beta \prime }_{i}x_{iy_{jt}t}\right)}{\sum_{j=1}^{J}exp\left({\beta \prime }_{i}x_{ijt}\right)}\right]$$

Under the assumption of heterogeneity in preferences (consumers are different in preferences), $\beta_{i}$ represent individual-specific coefficients associated with characteristics $x_{ij}$. Following Croissant [55], individual coefficients $\beta_{i}$ can be estimated by using the fitted method:

(2) $${\hat{\beta }}_{i}=\frac{\sum_{r}P_{ir}\beta_{r}}{\sum_{r}P_{ir}}$$

where $\beta_{r}$ represents the distribution of β at $r^{th}$ draw, and $P_{ir}$ is the estimated probability of simulated maximum likelihood with R draws. In this study, the coefficients of Equation (1) were estimated using a mixed logit approach that enables both fixed and random effects of the attributes in DCE, as suggested by Hole and Kolstad [56]. In particular, the price is fixed, while non-price attribute coefficients are assumed to be random effects ~N (μ, σ^2^) regarding the assumption of heterogeneity in consumer preferences [57].

Based on the estimated individual coefficients of the respondents’ preferences, WTP for tomato attributes can be computed as the ratio of estimated attribute coefficients (marginal utilities of the attributes) to estimated price coefficients (the marginal utility of price) in the choice model [58,59]. For instance, WTP for the “local” label from consumer “i” is calculated as follows:

(3) $${WTP}_{i}=-\frac{{\hat{\beta }}_{i}^{local}}{{\hat{\beta }}_{i}^{price}}$$

In the second stage, we employed linear models to measure the effects of explanatory variables on WTP for local food labels. Typically, a linear model can be specified as below:

(4) $$\eta_{i}=\rho h_{i}^{\prime }+\epsilon_{i}$$

where η is the amount that consumer “i” is willing to pay for “local” (WTP for a local label), ρ is a vector of coefficients, h is a vector of explanatory variables, and $\epsilon_{i}$ the error term that is independently and normally distributed with mean zero and variance $\sigma^{2}$. It is worth noting that we obtained WTP for the typical local label and WTP for Missouri Grown from Equation (3). Therefore, the WTP $\eta_{i}$ can be considered as observed, and Equation (4) can be applied to the two different designations of the local label. The estimates of ρ can be interpreted as the impacts of various variables h on the WTP for the corresponding local label. The explanatory variables h consist of potential factors that are extracted from the literature on local food: grocery shopping behavior (frequency), supporting attitudes to the local community, environmental and health attitudes of interest, and consumer characteristics (gender, age, race, education, household income, living location, and the presence of children under 17 years old).

We are interested in three unobservable factors in the assessment of the impacts of different attitudes, shopping behavior, and consumer characteristics on WTP for local labels. To do that, we adopt a structural equation model (SEM) to capture the causal relationships between latent or unobservable variables and observed variables [60]. The linear SEM has the following form:

(5) $$\begin{array}{l} \eta_{i}=\alpha v_{i}+\gamma {µ}_{i}+\epsilon_{i}\\ q_{i}=\Lambda {µ}_{i}+\delta_{i} \end{array}$$

In Equation (5), the structural part exhibits the relationship between the WTP for local labels η and “unobserved” attitude variables µ and observed variables (e.g., demographic variables), and the measurement part relates “unobserved” attitude variables µ and observed attitude-related instruments or indicators q.

Specifically, we utilize eleven statements of consumer attitudes towards local food purchase, collected from the survey, as observed indicators. These statements encompass two key areas: importance of sustainable local community development, environment protection, and improved health (six items), and agreement with sustainable local development, pro-environmental attitudes, and health concerns (five items). Using a factor analysis of these attitude-related indicators, we construct three latent variables within a reflective measurement model framework, where the observed variables (indicators) are assumed to reflect underlying latent constructs [61].

**Figure 1:**
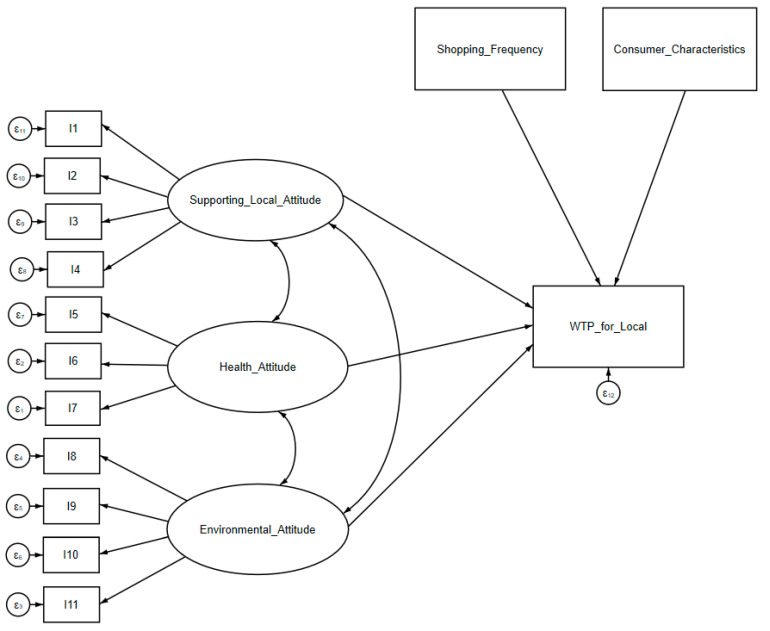
Proposed research model for Equation (5) (SEM).

The proposed latent variables (constructs) herein consist of supporting local attitudes, health attitudes regarding GMOs and pesticide residue, and general environmental attitudes (Figure 1). Subsequently, we examine the hypothesized causal effects of these latent variables on WTP for local labels in the structural part of Equation (5) [62]. It is worth noting that confirmatory factor analysis of the eleven attitude-related items is already performed within the context of the SEM framework. Compared to Equation (4), Equation (5) would be able to evaluate the complex relationship between the variables (both covariances and correlations) simultaneously [62]. Conventionally, reliability and validity analyses are provided for the assessment of the results from the SEM’s estimation. Equation (5) was estimated using the maximum likelihood method.

## 4. Results

### 4.1. Individual Consumer Preferences and WTP for Local Food

The first stage of analysis aims to obtain individual consumers’ preferences and WTPs for local attributes. A summary of the estimation results of the choice experiment for tomato purchase based on the mixed logit model is shown in Table 4. This table reports the means and the standard deviations of estimated coefficients of preferences for the tomato attributes. All the coefficients are statistically significant at the 5% level, indicating significant contributions of these attributes to consumer utility. The coefficients of the opt-out option and price are negative, indicating that a higher price or no purchase earns lower utility for consumers. The estimates of the attributes of organic, reduced pesticide use, local, Missouri Grown, and family producer are all positive, suggesting higher perceived utility for tomatoes with these attributes with respect to conventional, non-local tomatoes or those provided by large corporation producers. The standard deviations of all attributes except large family farms are significant, indicating the heterogeneity in preferences for the corresponding attributes over the sampled population. The different individuals own individual-specific parameter estimates [59].

By design, the coefficients of the local attributes (local and Missouri Grown labels) are estimated independently [63], with values of 0.096 and 0.442, respectively. The positive results indicate consumers typically prefer locally grown tomatoes over non-local ones. However, with the corresponding estimated SDs of 0.254 for local and 0.400 for Missouri Grown, WTPs for these two labels differ significantly across the sample. In other words, there are considerable differences among the individuals with respect to the effects of local and Missouri Grown attributes. The positive impacts of local labels on preferences and the heterogeneity of local attributes are consistent with the previous results on local tomatoes [63,64].

Individual WTPs for the tomato attributes from the choice experiment results are presented in Table 5. On average, the highest WTP is for “organic” at 66 cents/lb., followed by “Missouri Grown” 60 cents/lb., “large family” 31 cents/lb., “small & medium family” 28 cents/lb., “local” 13 cents/lb., and “50% reduced pesticide use” 10 cents/lb. Regarding local attributes, the sample means of WTP for tomatoes carrying a local label and a state logo are in line with previous studies (the WTP for local effect on tomatoes ranges 38–85 cents/lb.) [12,63]. The average WTP for Missouri Grown is substantially higher than for the local label, suggesting the difference between the local and the state brand due to a clearer definition of the state brand than the “local” [65]. Also, the high WTP for Missouri Grown implies a potential source for heterogeneity in preferences for local attributes. Consumers who support the state are willing to pay a higher premium for state-branded food than a local label (the label says locally grown or a local brand name, etc.), which we will discuss later in the second stage of analysis.

### 4.2. Influences of Consumer Health, Environmental, and Supporting Local Atitudes on WTP for Local Food 

The second stage is to examine the impacts of potential explanatory variables on WTP for local labels and evaluate the influences of health and environmental attitude variables. The predictors include attitude-related variables, grocery shopping frequency, gender, age, race, education, income, living location (urban), and having children under 17 years old. Before conducting the regression analysis, pairwise correlations of the independent variables were examined.

The correlation matrix shows moderate and high correlations between several attitude variables with coefficients of 0.50–0.86 (Supplementary Table S2), indicating potential multicollinearity issues in the model. To address multicollinearity, we calculated the Variance Inflation Factor (VIF) for every variable using the sample data (Supplementary Table S3). VIF scores range from 1.11 to 4.44, which are lower than the standard cutoff of 5, suggesting no evidence of a multicollinearity problem [66]. However, a collinearity test for the sample data reports a condition number of 32.35 (Supplementary Table S4), indicating another facet of the multicollinearity issue [67]. While the VIF result guarantees consistency and efficiency of estimates, the condition number suggests a different model structure for the attitude-related variables. Literally, the attitude-related variables of the study are inter-correlated in group by design. In this regard, based on the theoretically proposed model, Equation (5), we adopt the SEM, which includes supporting sustainable local community development, health, and environmental attitudes as latent variables to predict WTP for local labels, along with consumer characteristic variables.

Table 6 and Table 7 show important statistics for reliability and validity tests of the SEM approach. For reliability, we report the Cronbach’s α-coefficients of proposed latent factors as usual. All the Cronbach’s α are greater than the common threshold of 0.7 (Table 6), indicating acceptable internal consistency, i.e., how closely related a set of items are as a group. Thus, the attitude-related items of the questionnaire have good reliability for the scale of the study.

For validity assessment, we employ confirmatory factor analysis (CFA) to validate the relationship between the latent factors in terms of supporting local in economic sense, health concern, and environmental attitudes and their corresponding indicators. Conventionally, there are various fitting indices for the evaluation, including the Chi-square test (χ^2^), χ^2^/degree of freedom (df), Root Mean Square Error of Approximation (RMSEA), Standardized Root Mean Square Residual (SRMR), Comparative fit index (CFI), and Tucker–Lewis index (TLI) [68].

Table 7 reports the standardized factor loading and standard error (SE) of each attitude-related item and the model fit statistics using CFA. The results show that the value of χ^2^/df is approximately 3, the fit indices (CFI and TLI) are greater than 0.95, and the errors (RMSEA and SRMR) are smaller than 0.8, suggesting the measurement model has a good fit or the construct validity is acceptable, overall [62]. In addition, the composite reliability measures produced by the CFA for every latent factor are greater than 0.7, indicating the items in each group can consistently explain the corresponding latent variable [69].

Finally, the model results also inform acceptable levels of discriminant and convergent validity of the three variables supporting local, health concern regarding GMOs, pesticide residue on food, and general environmental attitude. With the average variance extraction (AVE) values are all above the threshold of 0.5 (Table 7), more than 50% of the variance in the indicators is explained by supporting local, health concern, and environment attitudes as required in the literature [70].

Following the best practices suggested by Cheung et al. [70] for assessing discriminant validity, the pairwise correlations among the three constructs in the model range from 0.3 to 0.5. These correlations are lower than the recommended threshold of 0.7 (indicating a shared variance of less than 49%), providing evidence that discriminant validity is not a concern given the estimated AVE values. To be precise, supporting local (in an economic sense), health consciousness regarding GMOs and pesticide residue on food, and general environmental attitudes are distinct constructs within the model.

Table 8 reports the estimation results of SEM, in which the dependent variables are WTP for local label and WTP for Missouri Grown, respectively. Overall, the model has an acceptable fit with χ^2^/df of 2.43 (<3), RMSEA 0.064 (<0.08), SRMR 0.042 (<0.08), and CFI 0.935 (>0.9) as previous studies [70,71].

## 5. Discussions

The effects of supporting farm/farmer attitudes are significantly positive on both WTP for local and Missouri Grown, indicating consumers who are supportive of local farms and farmers in Missouri are willing to pay a premium for locally labeled produce. The strong effects of supporting attitude, 0.235 on WTP for local food and 0.288 on WTP for Missouri Grown, indicate consumers with higher supporting local attitude are more likely to prefer local rather than non-local produce. This result is in line with previous studies [47,49]. Also, the results show little difference in the effects of supporting local farmer perspectives between local labels and the state logo.

Interestingly, the effect of health concerns on WTPs for local food and Missouri Grown is significantly negative. In this study, the health concerns are represented by GMOs and pesticide residue in food. Therefore, the results imply consumers more concerned about GMOs and pesticide residue in food would pay less for locally grown produce. An increase of one unit on the scale of health concerns would reduce payment by about 35 cents/lb. for typically local or Missouri Grown tomatoes. One possible reason is that organic is independent of local attributes in the study. Health concerned consumers are more likely to purchase organic tomatoes. Indeed, we examined the impacts of the health concerns on WTP for organic and found a significantly positive effect.

The effects of environmental attitude on the premiums for local and Missouri Grown are positive but small or insignificant. So, there is not enough evidence to conclude that consumers with pro-environmental attitudes are more likely to buy locally or pay more for locally grown produce. As mentioned previously, the impacts of environmental attitudes on consumer preferences and WTP for local food are debatable in the literature.

The relevant argument for possible impacts of environmental attitudes mostly comes from the link between local and sustainable concepts because local food is a kind of sustainable good due to less transportation or reduced emissions [51]. However, locally grown products do not necessarily have lower carbon footprints, as many more factors matter than just transportation for sustainability [72]. Further, our results indicate the effects of general environmental interests on WTP for “local” are not significant regardless of whether local is defined by distance or state-wise as indicated in this study.

Additionally, it would not be surprising that environmental attitudes did not affect the premiums for locals, as the labels are considered independent of tomato production methods. Similarly to the case of health concerns, we found environmental attitudes positively affect WTP for organic attributes. The findings imply that consumer preferences and WTP for local food would more likely be affected by personal health concerns rather than environmental attitudes regarding GMO and pesticide residue issues.

Among consumer behavior and characteristics, only race has a significantly negative effect on WTP for Missouri Grown. In particular, compared to non-Caucasian consumers, white people are willing to pay less, about 18 cents/lb. for Missouri Grown tomatoes. The effect of race on WTP for local food has little been studied in the literature. The findings of the study suggest more research is needed on race and other characteristics, which may play as control variables in measuring the impacts of farm supporting, health, and environmental attitudes. With the current sample, the effects of female, young, rural consumers are willing to pay more for local labels, but small. In other words, consumer characteristics might be sources for differences between WTP for local labels and the state logo, like Missouri Grown if present, rather than health and environmental attitudes.

The integration of consumer health and environmental attitudes into the development of local food systems has become increasingly important. Recent studies [73,74] have highlighted the potential of health and environmental labeling to guide consumers towards healthier and more sustainable choices. However, empirical evidence on how diverse health and environmental attitudes specifically influence consumer preferences for local food remains limited. Understanding how consumer health concerns and environmental interests influence their choices among local food, organic, non-GMO, and other healthy or sustainable food products is complex, particularly given the unobservable and heterogeneous nature of consumer attitudes and preferences.

This study aims to bridge this gap by enabling a more nuanced comparison between seemingly vague concepts related to local food, such as the distinctions between local labels and state logos, local versus organic food, and the interplay between economic and environmental motivations for supporting local food production. A significant strength of this study is the utilization of SEM to incorporate and analyze the influence of unobservable variables, such as personal attitudes, within the context of choice experiment data.

In previous studies, it is a significant challenge to incorporate these attitudes into the estimation of consumer WTP for food attributes, potentially requiring a reduction in the number of attributes and attribute levels to maintain model complexity. Furthermore, the direct use of consumer attitude indicators (the statements indicate supporting local food, health concerns regarding GMOs and pesticide residue, and environmental attitudes in this study) is not recommended in the choice model. These attitude statements may exhibit high levels of multicollinearity, potentially leading to unstable and unreliable model estimates. By applying SEM, we are able to identify the underlying motivations for different types of attitudes while acknowledging the potential intercorrelations among these attitudes.

This research is not without caveats. While WTP values are not able to be collected directly from consumers, the study obtains individual WTP estimates using a choice experiment framework which is grounded by strong economic theories and empirical methods to measure WTP for food attributes. It is worth noting that the accuracy of the estimates would increase for a higher number of choice situations in DCEs [58]. However, repeating a limited number of scenarios would be expected given time constraints and the complexity of online choice experiments. In this regard, the WTP estimates that are used in the regression analyses of the study were obtained from a DCE including nine scenarios. Another limitation of the study is related to the use of health and environmental claims. Since only a limited number of health and environmental attitude variables are included in the measurement model, the results of this study cannot be generalized for various health and environmental claims.

## 6. Conclusions

This study makes an attempt to examine the influences of diverse supporting local farmers, health concerns, and environmental interests on consumers’ WTP for local food, using the dataset from a survey on local food purchase behavior and a choice experiment involving tomato options conducted in Missouri. As expected, consumers who are more supportive of local farmers are willing to pay a higher premium for locally labeled produce. The influence mechanisms of health concerns and environmental interests on consumer WTP for local food were discussed, with the particular attention on potential difference between local and organic food.

The results show an increase in health concerns regarding GMOs and pesticide residue leads to lower WTP for local or Missouri Grown. The finding implies the effects of health concerns might be negative on local food purchases if another option like organic is presented to consumers. Considering various healthy and sustainable products on the food market, local food may be one of the choices for health and environmental benefits, but not the best regarding GMOs and pesticide residue compared to organic, for example. This finding supports the view that there may be publication bias against non-local results as the base can include organic and other sustainable attributes [19]. Also, the impacts of general environmental attitudes might be small compared to community support and health attitudes. In this regard, the finding is partly consistent with the discussion of environmental attitude impacts in the literature.

The information from our study can be used by farmers to inform production and sales decisions. Local farmers may earn consumer interest by promoting non-GMO, reduced, or free pesticide products, which can give their products a premium. Our research implies health and environmental attributes need to be specific or obvious to target specific consumer segments. For example, for consumers who are concerned about GMOs or pesticide residue in vegetables and fruits, local growers should explicitly present production methods such as organic, natural, heirloom, or at least free chemicals. Local growers might also inform consumers how their farming supports the community environment, such as reducing emissions in the neighborhood rivers, streams, or underground water.

Our findings also have implications for government programs that support local food. Consumers who are highly supportive of local farms, farmers, and communities are willing to buy local food and even pay more for local labels. Instead of looking to increase local food sales by promoting the health and environmental benefits of local food, these programs could consider increasing consumer awareness of local farms and farmers and promoting local movements using media and other informational channels. One of the popular options is to increase the availability and operation of FMs where consumers can meet and communicate directly with local farmers. This may increase sales and profitability for local producers because consumers can understand how to support them or how to understand the different benefits of farm produce. State sponsored marketing programs like Missouri Grown should take demographics like race into account to enhance the benefits of the Missouri Grown logo to farmers. However, more research is needed on sources for differences between a typical local label and the state brand.

Future research may consider the effects of health and environmental attitudes in diverse situations, e.g., expanding health attitudes or focusing on specific environmental interests for different products. While health concerns on fresh produce like vegetables or fruits might be directly related to GMOs and pesticide residue, there are lots of other health interests from eating them such as lowering the risks of obesity, heart disease, and stroke, improving mental health and healthy behaviors [75,76]. On the other hand, people might have different environmental priorities depending on context (home or vacation), environmental issues (e.g., global warming, climate change, recycling, green behaviors, etc.), and demographics (gender, education, race, etc.), what is known as heterogeneity in environmental attitudes [77,78,79]. Also, more research might be needed to consider different designations of local labels.

## Figures and Tables

**Table 1:** Example of a choice scenario in DCE of tomato purchases.

Option A	Option B	Option C
Organic	Conventional	50% Reduced pesticide
Not local or Missouri Grown	Local	Missouri Grown
Large family	Large corporation	Large family
$2.99/lb	$3.99/lb	$1.99/lb

**Table 2:** Sample statistics (N = 352).

Consumer Characteristics	Frequency	Percent
Gender		
Male	162	46.0%
Female	190	54.0%
Age		
18–24 years old	19	5.4%
25–34 years old	115	32.7%
35–44 years old	109	31.0%
45–54 years old	57	16.2%
55–64 years old	42	11.9%
65 years old and above	10	2.8%
Race		
Caucasian (white)	272	77.3%
African American	38	10.8%
Others	42	11.9%
Education		
High school or less	78	22.2%
Two-year college or associate degree	51	14.5%
Bachelor’s degree	150	42.6%
Graduate or professional degree	73	20.7%
Annual household income (USD)		
Less than $25,000	48	13.6%
$25,000–$50,000	119	32.1%
$50,000–$70,000	79	22.9%
$75,000–$100,000	48	14.3%
$100,000 and above	58	17.1%
Household living location		
Urban	118	33.5%
Suburban	138	39.2%
Rural	96	27.3%
Having children under 17 years of age		
No	177	50.3%
Yes	175	49.7%
Consumed local food in the past 12 months		
No	51	50.3%
Yes	301	85.5%
Shopping frequency for fresh fruits and vegetables		
Less than once a week or rarely	70	19.9%
Once a week	146	41.5%
More than once a week	136	38.6%

**Table 3:** Indicators of consumers’ attitudes toward local, health, and the environment.

Variables	Min	Max	Mean	SD ^(i)^
Importance ^(ii)^ of reducing water pollution	1	5	3.26	1.38
Importance of reducing soil erosion	1	5	3.24	1.34
Importance of supporting local farms and communities	1	5	3.78	1.16
Importance of supporting fair wages for farmers	1	5	3.58	1.24
Importance of limiting GMOs in food	1	5	3.13	1.47
Importance of minimizing pesticide residue	1	5	3.66	1.25
Agreement ^(iii)^ concerning chemicals (pesticides) and GMOs in personal diet	1	5	3.60	1.35
Agreement with buying environmentally friendly products because they are less polluting	1	5	3.95	0.95
Agreement with buying environmentally friendly products because they are healthier, safer, and better quality	1	5	4.03	0.97
Agreement with willingess to pay more to improve environmental protection	1	5	3.57	1.16
Agreement that the quality of life in my community depends on good water quality in local streams, rivers, and lakes	1	5	4.24	0.91

Notes: ^(i)^ SD represents the standard deviation. ^(ii)^ Importance statements are measured using a Likert scale (1–5, where 1 = “Not important at all” and 5 = “Important”). ^(iii)^ Agreement statements are measured using a Likert scale (1–5, where 1 = “Strongly disagree” and 5 = “Strongly agree”).

**Table 4:** Analysis of preferences for tomato attributes in the choice experiment.

Attributes	Coefficients	Error
Opt-out	−4.254 ***	0.143
Price	−0.740 ***	0.036
Organic	0.481 ***	0.034
50% reduced pesticide use	0.078 **	0.038
Local	0.096 **	0.042
Missouri Grown	0.442 ***	0.038
Small, medium family farm	0.210 ***	0.038
Large family farm	0.230 ***	0.037
SD (Organic)	0.654 ***	0.051
SD (50% Reduced pesticide use)	0.396 ***	0.070
SD (Local)	0.254 **	0.113
SD (Missouri Grown)	0.400 ***	0.071
SD (Small and medium family farm)	0.522 ***	0.060
SD (Large family farm)	0.002	0.353
**Model Statistics**		
Log-likelihood	−3266	
Wald χ^2^ (df)	214(6)	
Pr (>χ^2^)	0.000 ***	
AIC	6561	
Number of observations	3168	

Notes: Superscripts ** and *** indicate statistical significance at the 5 percent, and 1 percent levels, respectively.

**Table 5:** Individual willingness-to-pay (WTP) for tomato attributes (U.S.$/lb.) (n = 352).

Variables ^(^*^)^	Min	Max	Mean	SD
WTP for organic	−0.92	2.63	0.66	0.69
WTP for 50% reduced pesticide use	−0.60	1.03	0.10	0.28
WTP for local	−0.24	0.44	0.13	0.13
WTP for Missouri Grown	−0.25	1.54	0.60	0.31
WTP for small and medium family farm	−0.93	2.17	0.28	0.48
WTP for large family farm	0.31	0.31	0.31	0.00

Notes: (*) Individual consumer WTP is conceptualized as the monetary amount one is willing to pay for the attribute of interest with respect to its reference. Organic or 50% reduced pesticide is compared to the conventional production method. Local or Missouri Grown is compared to neither local nor Missouri Grown. Small and medium family or large family farm is compared to large corporation producer.

**Table 6:** Reliability analysis of latent variables.

Attitude-Related Items	Latent Variables	Cronback’s α
Importance of supporting local farms and communities	Supporting sustainable local community development	0.843
Importance of supporting fair wages for farmers		
Importance of limiting GMOs in food	Health concern regarding GMO, pesticide residue	0.756
Importance of minimizing pesticide residue		
Agreement with concerning about chemicals (pesticides) and GMOs in personal diet		
Agreement with buying environmentally friendly products because they are healthier, safer, and of better quality		
Importance of reducing water pollution	Environmental attitude	0.778
Importance of reducing soil erosion		
Agreement with buying environmentally friendly products because they are less polluting		
Agreement with willing to pay more to improve environmental protection		
Agreement with that the quality of life in my community depends on good water quality in local streams, rivers, lakes		

**Table 7:** Validity analysis of latent variables.

Variables	Measurement Items	Estimate	SE	CR	AVE
Supporting sustainable local community development	Importance of supporting local farms and communities	0.821 ***	0.026	805	0.735
	Importance of supporting fair wages for farmers	0.892 ***	0.024		
Health concerns regarding GMO, pesticide residue	Importance of limiting GMOs in food	0.711 ***	0.040	703	0.505
	Importance of minimizing pesticide residue	0.788 ***	0.037		
	Agreement concerning chemicals (pesticides) and GMOs in personal diet	0.625 ***	0.043		
Environmental attitude	Importance of reducing water pollution	0.926 ***	0.016	0.784	0.506
	Importance of reducing soil erosion	0.926 ***	0.016		
	Agreement with buying environmentally friendly products because they are less polluting	0.361 ***	0.049		
	Agreement with willing to pay more to improve environmental protection	0.424 ***	0.046		
**Model fit information**					
N	352				
χ^2^(df)	61.62(20) ***				
χ^2^/df	3.081				
RMSEA	0.077				
SRMR	0.046				
CFI	97.3				
TLI	95.1				

Notes: Superscripts *** indicate statistical significance at the 1 percent level.

**Table 8:** Estimation results of WTP for local attributes.

Independent Variables (Factors)	WTP for Local Label		WTP for Missouri Grown	
	Estimate	Std. Error	Estimate	Std. Error
***Attitudes and shopping behaviors***				
Supporting farm/farmer	0.235 **	0.119	0.288 ***	0.082
Health concern	−0.346 *	0.205	−0.359 ***	0.133
Environmental attitude	0.039	0.047	0.036	0.045
Grocery shopping frequency	−0.009	0.049	−0.022	0.068
***Consumer characteristics***				
Gender is female	0.046	0.046	0.081	0.052
Age (in years)	0.004	0.003	0.003	0.003
Race is white	−0.092	0.075	−0.177 ***	0.067
Highest education is bachelor or above	−0.076	0.047	−0.041	0.057
Annual household income	−0.015	0.021	−0.017	0.021
Living location (closeness to rural)	0.013	0.023	0.022	0.032
Having at least one child < 17 of age	0.034	0.048	0.083	0.052
Intercept	0.043	0.096	0.574	0.128
**Model fit statistics**				
Number of observations	352			
χ^2^(df)	194.61 (80)			
χ^2^/df	2.43			
RMSEA	0.064			
SRMR	0.042			
CFI	0.935			
TLI	0.884			
AIC	15198			

Notes: Superscripts *, **, and *** indicate statistical significance at the 10 percent, 5 percent, and 1 percent levels, respectively.

## Data Availability

The original contributions presented in this study are included in the article/Supplementary Material. Further inquiries can be directed at the corresponding author.

## Supplementary Materials

The following supporting information can be downloaded at: https://www.mdpi.com/article/10.3390/ijerph22020298/s1. Table S1: Discrete Choice Experiment for tomato purchase. Table S2: Correlation matrix of independent variables. Table S3: Variance Inflation Factor (VIF) of independent variables. Table S4: Collinearity test.
