# Supplementary material for: Consumers’ Health and Environmental Attitudes and Local Food Purchases
Source: Int J Environ Res Public Health. 2025 Feb 17;22(2):298. doi: 10.3390/ijerph22020298 (PMC11855125; doi:10.3390/ijerph22020298)
Supplement: Supplementary file 1 [file ijerph-22-00298-s001.zip › ijerph-3364091-supplementary.pdf]

SUPPLEMENTARY MATERIALS

**Table S1. Discrete Choice Experiment for tomato purchase**

| **Attributes** | **Levels** | | |
|---|---|---|---|
| | ***Level 1*** | ***Level 2*** | ***Level 3*** |
| ***Production method*** | Organic | 50% reduced pesticide(*) | Conventional |
| ***Label*** | Local | Missouri Grown(**) | Neither "Local" nor "Missouri Grown" |
| ***Farm type*** | Small & medium family | Large family | Large corporation |
| ***Price of tomatoes*** | $1.99/lb. | $2.99/lb. | $3.99/lb. |

*Notes:*
*(*) the 50% reduced pesticide techniques can be defined as the methods farmers use to reduce by half the pesticide amounts usually used in tomato cultivation.*
*(**) Missouri Grown is the state logo offered by Missouri Grown, an agricultural state marketing program of the Missouri Department of Agriculture, for the promotion of products grown, raised, or produced and processed in Missouri.*
*(***) The experimental design includes 9 random choice sets or scenarios where each scenario consists of 4 options: three alternative tomatoes and one opt-out ("None of these"). The statistical efficiency of this design is 98.6%.*

**Table S2. Correlation matrix of independent variables**

| | X1 | X2 | X3 | X4 | X5 | X6 | X7 | X8 | X9 | X10 | X11 | X12 | X13 | X14 | X15 | X16 | X17 | X18 | X19 |
|---|---|---|---|---|---|---|---|---|---|---|---|---|---|---|---|---|---|---|---|
| X1 | 1.00 | | | | | | | | | | | | | | | | | | |
| X2 | 0.86 | 1.00 | | | | | | | | | | | | | | | | | |
| X3 | 0.51 | 0.51 | 1.00 | | | | | | | | | | | | | | | | |
| X4 | 0.56 | 0.58 | 0.73 | 1.00 | | | | | | | | | | | | | | | |
| X5 | 0.37 | 0.38 | 0.40 | 0.41 | 1.00 | | | | | | | | | | | | | | |
| X6 | 0.49 | 0.49 | 0.50 | 0.51 | 0.51 | 1.00 | | | | | | | | | | | | | |
| X7 | 0.24 | 0.21 | 0.24 | 0.28 | 0.55 | 0.49 | 1.00 | | | | | | | | | | | | |
| X8 | 0.33 | 0.32 | 0.29 | 0.33 | 0.16 | 0.35 | 0.27 | 1.00 | | | | | | | | | | | |
| X9 | 0.28 | 0.26 | 0.32 | 0.41 | 0.29 | 0.37 | 0.40 | 0.63 | 1.00 | | | | | | | | | | |
| X10 | 0.40 | 0.39 | 0.23 | 0.38 | 0.17 | 0.20 | 0.16 | 0.49 | 0.47 | 1.00 | | | | | | | | | |
| X11 | 0.24 | 0.24 | 0.23 | 0.26 | 0.12 | 0.31 | 0.24 | 0.50 | 0.49 | 0.34 | 1.00 | | | | | | | | |
| X12 | 0.26 | 0.22 | 0.10 | 0.11 | 0.11 | 0.05 | 0.04 | 0.01 | 0.05 | 0.24 | 0.00 | 1.00 | | | | | | | |
| X13 | 0.08 | 0.07 | 0.19 | 0.22 | 0.24 | 0.16 | 0.15 | 0.08 | 0.12 | 0.01 | 0.07 | 0.00 | 1.00 | | | | | | |
| X14 | 0.06 | 0.05 | 0.19 | 0.09 | 0.13 | 0.21 | 0.23 | 0.09 | 0.09 | -0.08 | 0.17 | -0.02 | 0.15 | 1.00 | | | | | |
| X15 | -0.11 | -0.10 | 0.00 | -0.04 | -0.16 | -0.09 | -0.02 | 0.02 | 0.00 | -0.07 | 0.03 | 0.00 | 0.03 | 0.04 | 1.00 | | | | |
| X16 | 0.10 | 0.06 | -0.04 | -0.11 | -0.07 | -0.09 | -0.10 | 0.04 | -0.01 | 0.14 | 0.03 | 0.22 | -0.18 | 0.00 | -0.12 | 1.00 | | | |
| X17 | -0.16 | -0.21 | 0.01 | -0.09 | -0.19 | -0.09 | -0.14 | 0.01 | -0.02 | 0.00 | 0.06 | 0.13 | -0.13 | 0.07 | 0.09 | 0.25 | 1.00 | | |
| X18 | 0.09 | 0.16 | -0.07 | -0.07 | 0.10 | -0.06 | -0.11 | -0.09 | -0.04 | 0.07 | -0.08 | 0.22 | -0.08 | -0.06 | -0.20 | 0.25 | -0.06 | 1.00 | |
| X19 | 0.03 | -0.01 | 0.05 | 0.06 | 0.17 | -0.04 | 0.11 | -0.02 | 0.04 | 0.08 | -0.05 | 0.28 | 0.02 | -0.19 | -0.08 | 0.14 | 0.18 | 0.08 | 1.00 |

*Notes:*

- Attitude-related variables: X1 – X11, which are items I1 – I11 that are defined in Table 3, respectively.
- Grocery shopping frequency: X12, which is B2 in Table 3.
- Consumer characteristics: gender (X13), age (in years) (X14), race is white (X15), education is bachelor and above (X16), annual household income (X17), living location is urban (X18), and having at least one child under 17 years old (X19).

**Table S3. Variance Inflation Factor (VIF) of independent variables**

| Variables | VIF | Tolerance (1/VIF) |
|---|---|---|
| Reduce water pollution | 4.22 | 0.24 |
| Reduce soil erosion | 4.44 | 0.23 |
| Support local farms/ communities | 2.47 | 0.41 |
| Support fair wages for farmers | 2.87 | 0.35 |
| Limit GMOs in food | 1.97 | 0.51 |
| Minimize pesticide residue | 2.07 | 0.48 |
| Concerning chemicals and GMOs in food | 1.86 | 0.54 |
| Environmentally friendly products are less polluting | 2.06 | 0.49 |
| Environmentally friendly products are healthier, safer, and of better quality | 2.15 | 0.47 |
| Willing to pay more for environmental protection | 1.76 | 0.57 |
| The quality of life depends on good water quality in local streams, rivers, and lakes | 1.51 | 0.66 |
| Grocery shopping frequency | 1.31 | 0.76 |
| Gender is female | 1.15 | 0.87 |
| Age (in years) | 1.24 | 0.81 |
| Race is white | 1.11 | 0.90 |
| Highest education is bachelor or above | 1.30 | 0.77 |
| Annual household income | 1.32 | 0.76 |
| Living in urban | 1.28 | 0.78 |
| Having at least one child under 17 years old | 1.28 | 0.78 |

**Table S4. Collinearity test**

| | Eigenvalues | Condition index |
|---|---|---|
| 1 | 16.721 | 1.000 |
| 2 | 0.773 | 4.652 |
| 3 | 0.522 | 5.661 |
| 4 | 0.467 | 5.982 |
| 5 | 0.347 | 6.939 |
| 6 | 0.272 | 7.842 |
| 7 | 0.175 | 9.780 |
| 8 | 0.150 | 10.557 |
| 9 | 0.113 | 12.194 |
| 10 | 0.087 | 13.884 |
| 11 | 0.076 | 14.878 |
| 12 | 0.064 | 16.121 |
| 13 | 0.052 | 17.866 |
| 14 | 0.044 | 19.422 |
| 15 | 0.035 | 21.876 |
| 16 | 0.025 | 25.697 |
| 17 | 0.023 | 26.817 |
| 18 | 0.020 | 28.861 |
| 19 | 0.018 | 30.660 |
| 20 | 0.016 | 32.354 |
| Condition number | | 32.354 |
| Det(correlation matrix) | | 0.001 |
